# Proton Pump Inhibitors and Risk of Chronic Kidney Disease: Evidence from Observational Studies

**DOI:** 10.3390/jcm12062262

**Published:** 2023-03-15

**Authors:** Chieh-Chen Wu, Mao-Hung Liao, Woon-Man Kung, Yao-Chin Wang

**Affiliations:** 1 Department of Healthcare Information and Management, School of Health Technology, Ming Chuan University, Taoyuan 33300, Taiwan; 2 Department of Exercise and Health Promotion, College of Kinesiology and Health, Chinese Culture University, Taipei 11114, Taiwan; 3 Superintendent Office, Yonghe Cardinal Tien Hospital, New Taipei City 23148, Taiwan; 4 Division of Neurosurgery, Department of Surgery, Taipei Tzu Chi Hospital, Buddhist Tzu Chi Medical Foundation, New Taipei City 23142, Taiwan; 5 Department of Emergency, Min-Sheng General Hospital, Taoyuan 33044, Taiwan; 6 Graduate Institute of Injury Prevention and Control, College of Public Health, Taipei Medical University, Taipei 11031, Taiwan

**Keywords:** proton pump inhibitors, kidney disease, chronic kidney disease, acute kidney disease, meta-analysis

## Abstract

Previous epidemiological studies have raised the concern that the use of proton pump inhibitors (PPIs) is associated with an increased risk of kidney diseases. To date, no comprehensive meta-analysis has been conducted to assess the association between PPIs and the risk of chronic kidney disease (CKD). Therefore, we conducted a systematic review and meta-analysis to address the association between PPIs and CKD. The primary search was conducted in the most popular databases, such as PubMed, Scopus, and Web of Science. All observational studies evaluated the risk of CKD among PPI users, and non-users were considered for inclusion. Two reviewers conducted data extraction and assessed the risk of bias. Random-effect models were used to calculate pooled effect sizes. A total of 6,829,905 participants from 10 observational studies were included. Compared with non-PPI use, PPI use was significantly associated with an increased risk of CKD (RR 1.72, 95% CI: 1.02–2.87, *p* = 0.03). This updated meta-analysis showed that PPI was significantly associated with an increased risk of CKD. Association was observed in the same among moderate-quality studies. Until further randomized control trials (RCTs) and biological studies confirm these results, PPI therapy should not stop patients with gastroesophageal reflux disease (GERD). However, caution should be used when prescribing to patients with high-risk kidney disease.

## 1. Introduction

The global incidence and prevalence of kidney disease are increasing steadily, imposing a significant burden and becoming the eighth-leading cause of morbidity and mortality. Kidney disease is a global public health concern; it is projected to become the 5th most common cause of mortality globally by 2040 [1,2]. Acute and chronic kidney disease (CKD) are the two main types of kidney disease, and they are associated with substantial economic burden and deficits in quality of life. The incidence and prevalence of CKD vary globally [3]; however, the risk of progressive CKD is 60% higher among people living in the lowest socioeconomic quartile than in the highest quartile [4].

Proton pump inhibitors (PPIs) are one of the most prescribed medications for treating acid-related gastrointestinal disorders [5,6]. It is reported that the number of PPI prescriptions per year in the United States has doubled since 2000, with annual expenditures estimated at USD 13.5 billion [7,8]. A growing number of publications have raised concerns about the inappropriate use of PPIs (25–70%) [9,10,11]. Previous studies have reported an increased risk of hip fractures [12], community-acquired pneumonia [13], pancreatic cancer [5], and gastric cancer [14] among PPI users. Recent studies also have found a link to an increased risk of CKD among PPI users [15,16,17]. Although the biological mechanism of their association remains unclear, several possible mechanisms can explain the association between PPI use and CKD [18,19,20].

This current study aimed to provide a comprehensive and updated systematic review and meta-analysis to examine the association between PPI and CKD. Moreover, we also aimed to assess whether there is any difference in the association by region, study design, methodological quality, gender, and types of PPI.

## 2. Methods

**Study Protocol:** Our study was conducted and reported according to the Meta-analyses of Observational Studies in Epidemiology (MOOSE) checklist [21].

**Search Strategy:** We conducted a systematic search for observational studies in PubMed, Scopus, and Web of Science, up to 25 November 2022. The following combination keywords were used: *Proton pump inhibitor/s, and chronic kidney disease*. We did not restrict language in the initial search. The search strategy was developed with a discussion with experts who have 5 years of experience in conducting systematic reviews and meta-analyses. In addition, a manual search was conducted through the reference lists of previously published reviews and meta-analyses to identify missing studies.

**Study Eligibility:** We considered all types of observational studies that evaluated the association between PPI use and the risk of CKD. Studies were included if they were (i) published in English, (ii) provided clear information about PPI users and inclusion criteria for CKD, and (iii) provided sufficient information to calculate a pooled effect size.

Studies were excluded if they were review articles, reports, animal research, conference abstracts, editorials, case reports, or studies without a comparator group. Two authors (CCW and MHL) independently screened all titles, abstracts, and full texts of all included studies. Any discrepancy during the study screening process was resolved through discussion with a third author.

**Data Extraction:** The same two authors developed the data extraction form to collect relevant information from selected full-text articles. The following information was extracted from selected studies: (i) basic information: author name, publication year, and origin; (ii) population: sample size, data source, age, and gender; (iii) methods: study design, inclusion and exclusion criteria, study duration, follow-up time, and adjustments for confounding factors; (iv) outcome: effect sizes with 95% confidence intervals (CIs).

**Assessment of Risk Bias:** We assessed the quality of included studies using the Newcastle–Ottawa Scale recommended by the Cochrane library [22]. It evaluates the quality of the nonrandomized studies based on the patient selection, comparability, and ascertainment of either the exposure or outcome of interest. A star system is used to judge the study quality with a maximum of 9 stars (4 stars for selection, 2 stars for comparability, and 3 stars for outcome). A study with 9 stars was classified as high quality, 7–8 stars as moderate, and <7 stars as low quality [5,12,23].

**Statistical Analysis**: The statistical analysis was performed using Comprehensive Meta-analysis (CMA) software. The pooled risk ratios (RR) with 95% confidence intervals were estimated using a random effects model based on the DerSimonian–Laird method. We drew forest plots to depict the visual interpretation of pooled estimates with 95% CIs. The Cochran Q test and I^2^ statistic were calculated to assess the degree of heterogeneity among studies. The significance level for the effect size was considered at *p* < 0.05.

## 3. Results

**Figure 1:**
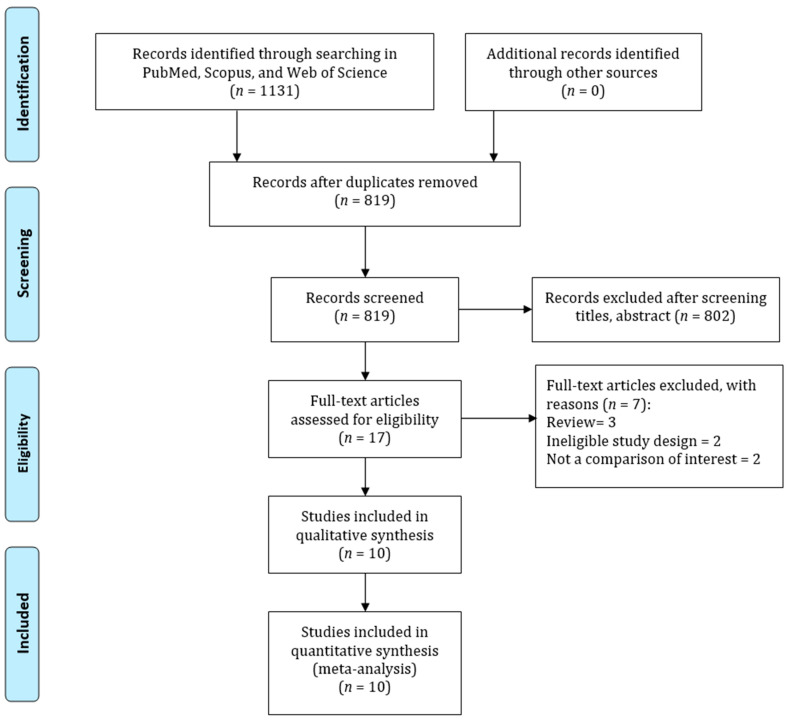
PRISMA guidelines for searching strategy of the association between PPI and CKD risk.

**Study Identification**: Figure 1 shows the flowchart of the study selection process in this study. The electronic databases search yielded 1131 articles; 312 of these were excluded for duplication. Moreover, 802 articles were further excluded due to irrelevant titles or abstracts. Thus, 17 full-text articles were screened, and 7 studies were further excluded due to being reviews, not a comparison of interest, and having ineligible study designs. Finally, 10 studies were included in this meta-analysis [15,24,25,26,27,28,29,30,31,32].

**Study Characteristics and Quality Assessment**: Table 1 shows the characteristics of the included studies. Among the 10 articles included in this study, 7 were cohort studies, and 3 were case-control studies. The range of the publication period was 2016 to 2022. Six studies were conducted in western countries, and four were from Asian countries. The sample size range of included studies was between 18,504 and 5,414,695. All the included studies used standard protocols to identify PPI users and CKD. The average NOS score was 8, with an interquartile range (IQR) of 7–9.

**Proton Pump Inhibitor and Chronic Kidney Disease**: Ten studies examined the risk of CKD among PPI users. PPI use was significantly associated with an increased risk of CKD compared to non-PPI users. The pooled RR was 1.72 (95% CI: 1.02–2.87, *p* = 0.03), with a significant heterogeneity among studies (Q = 8730.48, *p* < 0.001, I^2^ = 99.88%)

**Subgroup Analysis**: We also conducted comprehensive subgroup analyses of the included 10 studies based on study design, region, methodological quality, gender, comorbidities, comedication, and types of PPI use (Table 2).

Seven cohort and three case-control studies evaluated the risk of CKD among PPI users. The adjusted pooled analysis of the seven cohort studies showed an increased risk of CKD among PPI users compared to non-PPI users (RR: 1.69, 95% CI: 0.85–3.35, *p* = 0.13). The pooled RR of CKD among PPI users for case-control studies was 1.57 (95% CI: 1.20–2.05, *p* = 0.001). The heterogeneity among the studies were Q = 7784.31, *p* < 0.001, and I^2^ = 99.91% and Q = 83.62, *p* < 0.001, and I^2^ = 97.60, respectively.

Six studies from Western countries examined the impact of PPI therapy on the risk of CKD. The overall pooled RR was 1.28 (95% CI: 1.17–1.40, *p* < 0.001), with significant heterogeneity among the studies (Q = 66.03, *p* < 0.001, I^2^ = 90.91%). Moreover, the pooled RR for studies from Asia was 2.25 (95% CI: 0.74–6.81, *p* = 0.14), with significant heterogeneity among studies (Q = 4858.83, *p* = 0.001, I^2^ = 99.93%).

**Figure 2:**
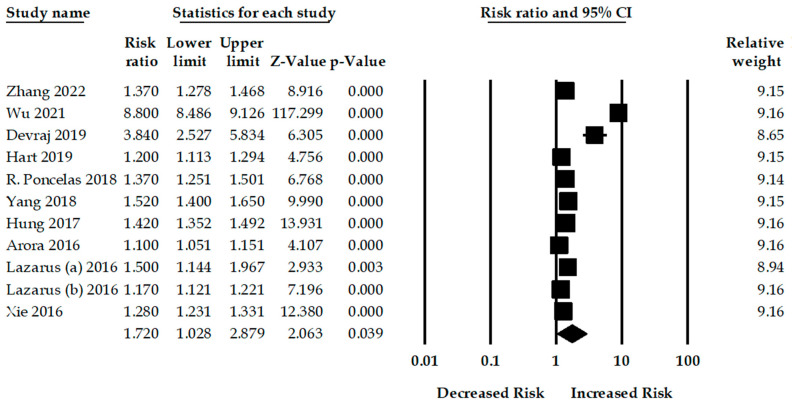
Association between PPI use and risk of chronic kidney disease [15,23,24,25,26,27,28,29,30,31].

The overall pooled RRs for the risk of CKD for high- and moderate-quality methodologies were 1.35 (95% CI: 1.23–1.49, *p* < 0.001, number of studies, *n* = 4) and 1.97 (95% CI: 0.95–4.07, *p* = 0.06, *n* = 6), respectively. Three studies evaluated the risk of CKD among male PPI users, and the adjusted pooled RR was 1.14 (95% CI: 1.01–1.28, *p* = 0.03). Moreover, four studies assessed the risk of CKD among female PPI users, and the adjusted pooled RR was 0.95 (95% CI: 0.63–1.42, *p* = 0.80) (Figure 2).

The studies assessed the risk of CKD with esomeprazole; the pooled RR was 1.32 (95% CI: 1.23–1.42, *p* < 0.001), with non-significant heterogeneity (Q = 0.82, *p* = 0.66, I^2^ = 0). The pooled RR for studies using rabeprazole and esomeprazole were 1.50 (95% CI: 1.20–1.87, *p* < 0.001, *n* = 2), 1.53 (95% CI: 1.24–1.89, *p* < 0.001, *n* = 2).

## 4. Sensitivity Analysis

The findings of this study had high heterogeneity (I^2^ = 99.88%, *p* < 0.001); therefore, we conducted a sensitivity analysis to observe any change in the findings. In order to evaluate the magnitude of the overall impact of each included study on CKD risk, a sensitivity analysis was performed by excluding studies one by one. However, this study did not observe any difference in overall effect size and heterogeneity among studies (Table 3).

**Figure 3:**
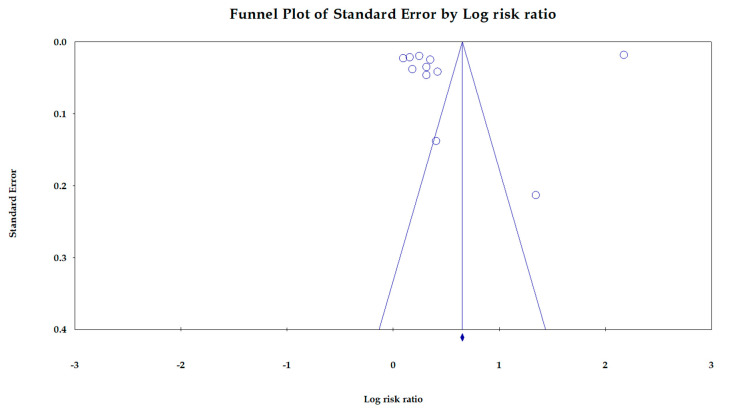
Funnel plot for the association between PPI use and the risk of CKD.

**Publication Bias**: Figure 3 shows the funnel plot of the association between PPI use and the risk of CKD. This Egger’s regression plot indicates no significant publication bias (*p* = 0.53).

## 5. Discussion

This systematic review with meta-analysis provides a comprehensive estimation of the association between PPI use and the risk of CKD. The findings of this study are based on previously published observational studies. The present study calculated adjusted pooled effect sizes using a random effects model from 10 studies, which included a total of 6,829,905 participants. The results showed that PPI use was significantly associated with a high CKD risk of 72%. These findings are supported by previously published meta-analyses [33,34,35,36], which showed PPI use increased the risk of CKD.

The biological mechanism underlying the positive association between PPI use and CKD risk is unclear. A previous study suggested that infection and inflammations could partially contribute to the development of CKD among PPI users [16]. Previous evidence also revealed that PPIs have a potential influence on the gut microbiota, which are responsible for intestinal microbial imbalance, thus increasing the risk of enteric infection [37,38,39]. Imhann et al. [40] and Jackson et al. [41] demonstrated that the rates of Enterobacteriaceae and Streptococcaceae increase in the gut among PPI users. PPIs escalate the accumulation of gut-derived uremic toxins, which ultimately induce CKD progression [42]. PPI use is associated with an increased risk of developing hypomagnesemia [43,44]. Evidence indicates that magnesium depletion leads to the induction of CKD. Previous studies also reported that PPI could cause inflammation and tubulointerstitial damage, which could ultimately lead to CKD [45,46,47].

Our subgroup analyses showed that the risk of CKD among PPI users was higher in Asian people than in Western people. Although an insignificant association was observed among Asians, it may be due to a small number of studies. Previous epidemiological studies reported that clinical, metabolic, socioeconomic, and behavioral factors could contribute to a higher risk of CKD in Asian people compared to others [48,49,50]. However, in Western countries, people are also at high risk of developing CKD because of the higher prevalence of diabetes and hypertension [51]. Other risk factors, such as cardiovascular disease, smoking status, and obesity, may affect ethnic groups differently, but this has not been tested due to a lack of data [3,52]. Our subgroup analyses also showed that omeprazole, rabeprazole, and esomeprazole are significantly associated with an increased risk of CKD. These PPIs disturb the balance of the pH level in the gastrointestinal tract, thereby reducing absorption mediated by TRPM6 and TRPM7 transporters [53]. Our findings also showed that male patients had a higher risk than female patients. As in other diseases, gender is a fundamental factor of CKD patients because males and females differ in renal physiology, complications, signs, and symptoms of CKD.

## 6. Strengths and Limitations

To our knowledge, this is the largest collection of observational studies on the assessment of PPI use for the risk of CKD, representing more than 10 studies compared with a comprehensive meta-analysis conducted previously. Unlike most recent meta-analyses, which do not have broad subgroup and sensitivity analysis, our study has more comprehensive search methods and analyses. This study also has some limitations. First, this meta-analysis was limited by the quality of the included studies. The pooled evidence from observational studies (cohort and case-control studies) cannot provide an interpretation regarding causation. Second, the findings of this study are prone to selection bias, confounding bias, and exaggeration of associations. However, we considered only the adjusted effect size to calculate a pooled effect size. Third, the study designs, data materials, statistical approaches, duration, and quality of included studies varied. Fourth, no information on the dose and duration of PPI use was available in included studies. Therefore, we were unable to provide the dose and duration effect on CKD risk. Finally, we are unable to classify the risk of CKD based on various stages due to a lack of data.

## 7. Conclusions

This systematic review and meta-analysis showed that PPI use was associated with an increased risk of CKD. The findings of our study could contribute to a more comprehensive understanding of CKD risk among PPI users. Our study findings highlight the need for early intervention among patients at high risk of CKD and for continuous monitoring of patients with PPI.

## Figures and Tables

**Table 1:** Shows the basic characteristics of studies included to evaluate the association between PPI and CKD.

Author	Year	Country	Study Design	Study Participant	Age (Year)	Gender (Male)	Inclusion Criteria for CKD	Adjustment	NOS
Zhang et al. [15]	2022	China	Cohort	462,421	58.89	45.3	ICD	Age, sex, smoking, alcohol consumption, BMI, physical activity, diabetes hypertension, hyperlipidemia, GORD, NSAIDs	9
Wu et al. [16]	2021	China	Cohort	5,414,695	Range	N/A	ICD	Age, sex	7
Devraj et al. [17]	2019	USA	C-C	18,504	46.3	48.2	ICD	Age, sex, BMI, race, smoking, alcohol, comorbidities	7
Hart et al. [18]	2020	USA	Cohort	177,935	51.1	38.7	ICD	Age, sex, BMI, smoking, alcohol, hypertension	9
Rodríguez-Poncelas et al. [19]	2018	Spain	Cohort	46,541	41.23	51.2	ICD	Age, gender, diabetes, obesity, blood pressure, hypertension, cholesterol, chronic disease	9
Yang et al. [20]	2018	UK	Cohort	29,970	59.1	59.5	ICD	Age, sex, hypertension, gout, IHD CVA, CHF, PAD, region	9
Hung et al. [24]	2017	Taiwan	C-C	33,408	Range	58.6	ICD	Age, sex, diabetes, hypertension	7
Arora et al. [21]	2016	USA	C-C	99,269	N/A	N/A	ICD	Age, sex, COPD, diabetes, hypertension	7
Lazarus (a) et al. [22]	2016	USA	Cohort	104,820	62.8	42.5	ICD	Age, sex, diabetes, diuretic use	8
Lazarus (b) et al. [22]	2016	USA	Cohort	248,751	50.0	43.2	ICD	Age, sex, CCI, DM, other lipid-lowering agents	8
Xie et al. [23]	2016	USA	Cohort	193,591	56.85	93.4	ICD	Age, sex, race, diabetes, hypertension, cardiovascular disease, GORD, chronic lung disease, ulcer disease	8

Note: CVA, cerebrovascular disease; IHD, ischemic heart disease; PAD, peripheral arterial disease; CHF, congestive heart failure, GORD = gastroesophageal reflux disease, N/A = not applicable; NOS, Newcastle–Ottawa Scale.

**Table 2:** Subgroup analysis for the association between PPI and CKD.

Study	No. of Studies	Pooled Estimates		Test of Heterogeneity		
		RR (95% CI)	*p*-Value	Q Value	*p*-Value	I^2^ (%)
All Studies	10	1.72 (1.02–2.87)	0.03	8730.48	<0.001	99.88
Study Design						
Cohort	7	1.69 (0.85–3.35)	0.13	7784.31	<0.001	99.91
Case-control	3	1.57 (1.20–2.05)	0.001	83.62	<0.001	97.60
Region						
Western	6	1.28 (1.17–1.40)	<0.001	66.03	<0.001	90.91
Asian	4	2.25 (0.74–6.81)	0.14	4858.83	0.001	99.93
Methodological Quality						
High	4	1.35 (1.23–1.49)	<0.001	17.70	<0.001	83.05
Moderate	6	1.97 (0.95–4.07)	0.06	8337.98	<0.001	99.92
Gender						
Male	3	1.14 (1.01–1.28)	0.03	21.73	<0.001	90.80
Female	4	0.95 (0.63–1.42)	0.80	111.94	<0.001	97.32
Comorbidities						
Hypertension	5	1.38 (0.95–1.99)	0.08	555.15	<0.001	99.27
Diabetes	4	1.45 (1.27–1.65)	<0.001	18.02	<0.001	83.35
Comedication						
NSAIDs	3	0.82 (0.45–1.51)	0.54	317.00	<0.001	99.36
Type of PPIs						
Lansoprazole	3	3.82 (0.40–36.46)	0.24	2953.10	<0.001	99.93
Omeprazole	3	1.32 (1.23–1.42)	<0.001	0.82	0.66	0
Pantoprazole	2	4.13 (0.49–34.21)	0.18	314.54	<0.001	99.68
Rabeprazole	2	1.50 (1.20–1.87)	<0.001	0.02	0.86	0
Esomeprazole	2	1.53 (1.24–1.89)	<0.001	4.31	0.03	76.83

**Table 3:** Sensitivity analysis for the association between PPI and CKD risk.

Excluded Study	Pooled Estimates		Test of Heterogeneity		
	RR (95% CI)	*p*-Value	Q Value	*p*-Value	I^2^ (%)
All Studies	1.72 (1.02–2.87)	0.03	8730.42	<0.001	99.88
Arora et al. [30]	1.80 (1.02–3.17)	0.04	8066.26	<0.001	99.88
Devraj et al. [26]	1.59 (0.93–2.73)	0.09	8719.87	<0.001	99.89
Hart et al. [27]	1.78 (1.02–3.10)	0.04	8573.17	<0.001	99.89
Huang et al. [32]	1.75 (0.98–3.12)	0.05	8569.41	<0.001	99.89
Lazarus (a) et al. [15]	1.74 (1.01–2.99)	0.04	8727.31	<0.001	99.89
Lazarus (b) et al. [15]	1.78 (1.00–3.18)	0.04	8123.88	<0.001	99.88
R. Poncelas et al. [28]	1.76 (1.01–3.05)	0.04	8676.41	<0.001	99.89
Wu et al. [25]	1.34 (1.24–1.45)	<0.001	128.22	<0.001	92.98
Xie et al. [31]	1.77 (0.98–3.19)	0.05	8226.78	<0.001	99.89
Yang et al. [29]	1.74 (1.00–3.03)	0.05	8698.44	<0.001	99.89
Zhang et al. [24]	1.76 (1.00–3.07)	0.04	8634.15	<0.001	99.89
